# Supplementary material for: Insomnia, Time Perspective, and Personality Traits: A Cross-Sectional Study in a Non-Clinical Population
Source: Int J Environ Res Public Health. 2022 Sep 3;19(17):11018. doi: 10.3390/ijerph191711018 (PMC9517905; doi:10.3390/ijerph191711018)
Supplement: Supplementary file 1 [file ijerph-19-11018-s001.zip › ijerph-1861267-supplementary.pdf]

| Ss | Sex | Age | PN | PH | F | PP | PF | DBTP | ISI | rMEQ | AG | CON | ES | EX | OP | Education | Shift-work | Nigh-SW |
|---|---|---|---|---|---|---|---|---|---|---|---|---|---|---|---|---|---|---|
| 1 | 2 | 24 | 2,70 | 3,00 | 3,08 | 4,00 | 2,22 | 1,76 | 6 | 17 | 3,00 | 3,00 | 3,00 | 4,50 | 4,50 | 4 | 0 | 0 |
| 2 | 1 | 26 | 2,40 | 3,60 | 3,77 | 4,00 | 2,00 | 0,98 | 12 | 17 | 4,00 | 2,50 | 2,50 | 1,50 | 3,50 | 3 | 0 | 0 |
| 3 | 1 | 20 | 2,90 | 3,20 | 3,00 | 3,78 | 3,11 | 2,38 | 7 | 12 | 2,50 | 3,00 | 3,50 | 4,00 | 2,50 | 3 | 0 | 0 |
| 4 | 2 | 42 | 3,20 | 2,00 | 3,38 | 4,33 | 2,56 | 2,60 | 5 | 18 | 1,50 | 5,00 | 3,00 | 4,50 | 3,00 | 3 | 1 | 2 |
| 5 | 2 | 61 | 3,80 | 2,40 | 3,46 | 1,44 | 2,67 | 4,16 | 2 | 17 | 2,50 | 4,50 | 3,00 | 3,00 | 2,00 | 4 | 0 | 0 |
| 6 | 1 | 44 | 1,80 | 1,67 | 3,54 | 3,33 | 2,11 | 2,68 | 0 | 21 | 3,00 | 3,00 | 5,00 | 3,00 | 3,00 | 3 | 0 | 0 |
| 7 | 2 | 26 | 3,90 | 2,60 | 3,62 | 3,67 | 3,11 | 3,02 | 6 | 13 | 3,00 | 4,00 | 1,00 | 2,00 | 4,00 | 3 | 0 | 0 |
| 8 | 2 | 35 | 3,00 | 2,80 | 3,62 | 4,11 | 2,67 | 2,02 | 10 | 16 | 4,00 | 4,00 | 1,50 | 3,00 | 2,50 | 2 | 0 | 0 |
| 9 | 1 | 56 | 3,60 | 3,07 | 3,69 | 3,67 | 2,89 | 2,51 | 7 | 23 | 4,00 | 4,00 | 3,50 | 5,00 | 4,00 | 3 | 1 | 2 |
| 10 | 1 | 39 | 3,20 | 3,47 | 3,38 | 4,22 | 3,11 | 2,21 | 14 | 11 | 3,00 | 3,50 | 3,00 | 3,50 | 4,00 | 4 | 0 | 0 |
| 11 | 2 | 67 | 3,60 | 3,73 | 3,54 | 4,56 | 4,33 | 3,32 | 6 | 19 | 5,00 | 3,50 | 3,50 | 3,00 | 2,50 | 3 | 0 | 0 |
| 12 | 1 | 48 | 3,10 | 2,73 | 3,15 | 3,67 | 2,44 | 2,27 | 4 | 18 | 4,50 | 4,50 | 2,50 | 2,50 | 3,50 | 3 | 0 | 0 |
| 13 | 2 | 35 | 2,90 | 2,67 | 4,15 | 2,78 | 2,56 | 2,62 | 7 | 18 | 3,00 | 5,00 | 2,50 | 4,00 | 3,50 | 4 | 0 | 0 |
| 14 | 2 | 53 | 3,70 | 3,00 | 2,69 | 3,89 | 3,78 | 3,36 | 10 | 13 | 3,00 | 3,50 | 2,00 | 5,00 | 3,00 | 1 | 0 | 0 |
| 15 | 2 | 48 | 2,40 | 2,47 | 3,38 | 3,89 | 2,00 | 1,84 | 2 | 21 | 5,00 | 4,50 | 3,50 | 3,50 | 1,50 | 2 | 1 | 2 |
| 16 | 2 | 51 | 4,30 | 3,93 | 4,15 | 4,11 | 3,44 | 3,09 | 3 | 20 | 3,00 | 5,00 | 1,50 | 5,00 | 5,00 | 2 | 0 | 0 |
| 17 | 2 | 35 | 3,10 | 3,13 | 2,77 | 2,78 | 2,67 | 2,85 | 3 | 21 | 3,00 | 5,00 | 1,50 | 5,00 | 5,00 | 3 | 0 | 0 |
| 18 | 2 | 51 | 2,70 | 2,53 | 3,46 | 3,22 | 2,67 | 2,45 | 0 | 17 | 2,50 | 4,00 | 3,00 | 5,00 | 4,00 | 1 | 0 | 0 |
| 19 | 2 | 22 | 3,10 | 3,53 | 2,92 | 3,89 | 3,11 | 2,39 | 12 | 9 | 3,50 | 3,00 | 1,00 | 3,50 | 3,50 | 3 | 1 | 2 |
| 20 | 1 | 25 | 3,20 | 3,67 | 3,46 | 3,67 | 2,56 | 1,97 | 9 | 12 | 3,00 | 3,50 | 3,00 | 3,00 | 5,00 | 4 | 0 | 0 |
| 21 | 1 | 58 | 2,80 | 3,00 | 2,69 | 4,44 | 2,44 | 2,04 | 0 | 16 | 3,00 | 3,50 | 2,50 | 3,50 | 4,00 | 2 | 0 | 0 |
| 22 | 1 | 28 | 2,80 | 2,80 | 3,77 | 3,67 | 2,33 | 1,88 | 4 | 17 | 4,50 | 3,50 | 4,00 | 4,50 | 3,00 | 1 | 1 | 2 |
| 23 | 2 | 54 | 2,10 | 2,60 | 3,23 | 4,00 | 2,11 | 1,74 | 0 | 18 | 3,00 | 5,00 | 3,00 | 4,00 | 1,50 | 2 | 0 | 0 |
| 24 | 1 | 61 | 2,60 | 3,20 | 3,92 | 3,44 | 2,22 | 1,67 | 4 | 20 | 4,50 | 3,50 | 2,00 | 5,00 | 2,50 | 2 | 0 | 0 |
| 25 | 2 | 24 | 2,50 | 3,20 | 3,77 | 4,22 | 2,44 | 1,37 | 4 | 19 | 3,50 | 3,50 | 3,50 | 3,50 | 3,00 | 4 | 0 | 0 |
| 26 | 1 | 44 | 3,00 | 3,00 | 3,85 | 3,44 | 3,44 | 2,66 | 4 | 20 | 3,50 | 4,50 | 3,50 | 3,50 | 3,50 | 3 | 0 | 0 |
| 27 | 2 | 42 | 2,10 | 2,93 | 3,31 | 3,33 | 2,89 | 2,23 | 2 | 17 | 3,50 | 4,50 | 4,00 | 5,00 | 1,00 | 2 | 0 | 0 |
| 28 | 2 | 63 | 2,40 | 2,80 | 3,69 | 3,78 | 3,22 | 2,27 | 4 | 20 | 4,00 | 4,50 | 2,00 | 5,00 | 4,00 | 2 | 0 | 0 |
| 29 | 2 | 55 | 2,00 | 2,87 | 3,77 | 4,33 | 2,56 | 1,52 | 5 | 20 | 5,00 | 5,00 | 2,50 | 3,50 | 2,00 | 2 | 0 | 0 |
| 30 | 2 | 44 | 2,70 | 2,60 | 3,38 | 3,22 | 2,11 | 2,21 | 3 | 19 | 4,50 | 5,00 | 3,00 | 5,00 | 3,50 | 2 | 0 | 0 |
| 31 | 2 | 38 | 3,40 | 3,40 | 3,92 | 3,33 | 3,33 | 2,71 | 24 | 13 | 2,50 | 3,50 | 3,50 | 4,00 | 2,50 | 4 | 0 | 0 |
| 32 | 1 | 54 | 2,20 | 2,60 | 3,77 | 3,89 | 3,33 | 2,38 | 6 | 19 | 4,00 | 4,00 | 3,00 | 2,50 | 2,50 | 2 | 0 | 0 |

| | | | | | | | | | | | | | | | | | | |
|---|---|---|---|---|---|---|---|---|---|---|---|---|---|---|---|---|---|---|
| 33 | 1 | 26 | 3,70 | 3,47 | 3,38 | 4,00 | 3,78 | 3,03 | 19 | 13 | 4,50 | 5,00 | 3,00 | 4,50 | 3,00 | 3 | 0 | 0 |
| 34 | 2 | 27 | 1,80 | 2,33 | 3,85 | 3,56 | 2,11 | 1,99 | 4 | 17 | 3,50 | 5,00 | 3,50 | 2,00 | 3,00 | 4 | 1 | 2 |
| 35 | 1 | 24 | 2,40 | 3,27 | 3,23 | 3,11 | 2,44 | 2,07 | 9 | 12 | 3,50 | 4,00 | 3,00 | 2,50 | 4,50 | 4 | 0 | 0 |
| 36 | 1 | 64 | 3,00 | 2,73 | 3,69 | 3,89 | 3,78 | 2,87 | 2 | 22 | 3,50 | 5,00 | 5,00 | 2,50 | 1,50 | 2 | 0 | 0 |
| 37 | 2 | 56 | 4,40 | 2,67 | 3,69 | 3,78 | 3,89 | 3,74 | 4 | 19 | 2,50 | 3,50 | 1,00 | 2,00 | 3,00 | 1 | 0 | 0 |
| 38 | 2 | 26 | 4,10 | 4,13 | 3,46 | 2,78 | 3,11 | 3,30 | 9 | 10 | 2,50 | 2,00 | 1,00 | 3,00 | 2,00 | 3 | 0 | 0 |
| 39 | 1 | 28 | 2,90 | 2,60 | 3,15 | 3,11 | 2,67 | 2,62 | 15 | 12 | 3,00 | 3,50 | 2,00 | 2,50 | 2,00 | 2 | 0 | 0 |
| 40 | 1 | 25 | 3,70 | 2,60 | 3,69 | 4,00 | 2,33 | 2,43 | 7 | 18 | 3,00 | 4,00 | 2,50 | 2,50 | 3,00 | 3 | 0 | 0 |
| 41 | 2 | 30 | 2,20 | 2,20 | 4,46 | 4,00 | 1,67 | 1,89 | 7 | 19 | 4,00 | 4,00 | 3,00 | 1,50 | 3,50 | 3 | 0 | 0 |
| 42 | 2 | 48 | 3,30 | 3,07 | 4,00 | 3,56 | 2,33 | 2,07 | 1 | 18 | 3,00 | 3,50 | 2,50 | 3,00 | 1,50 | 2 | 0 | 0 |
| 43 | 1 | 28 | 2,00 | 2,47 | 4,00 | 3,78 | 2,33 | 1,85 | 3 | 13 | 3,50 | 3,00 | 3,50 | 2,00 | 3,50 | 3 | 1 | 2 |
| 44 | 2 | 45 | 3,00 | 2,47 | 3,77 | 2,56 | 1,44 | 2,72 | 5 | 21 | 4,50 | 4,00 | 3,50 | 3,00 | 2,00 | 4 | 0 | 0 |
| 45 | 2 | 50 | 2,70 | 2,80 | 4,31 | 3,67 | 1,67 | 1,66 | 8 | 20 | 4,50 | 4,50 | 2,50 | 2,50 | 3,00 | 2 | 0 | 0 |
| 46 | 2 | 30 | 3,30 | 4,13 | 4,00 | 3,22 | 3,11 | 2,52 | 5 | 14 | 4,00 | 3,00 | 3,00 | 4,00 | 4,00 | 3 | 0 | 0 |
| 47 | 2 | 40 | 3,50 | 3,80 | 4,69 | 3,22 | 3,00 | 2,65 | 6 | 19 | 3,50 | 3,50 | 3,50 | 3,50 | 3,00 | 4 | 1 | 2 |
| 48 | 1 | 58 | 2,20 | 1,60 | 2,92 | 3,44 | 2,11 | 2,87 | 8 | 15 | 4,00 | 3,00 | 4,00 | 2,50 | 2,50 | 2 | 1 | 1 |
| 49 | 2 | 35 | 3,00 | 4,13 | 3,38 | 4,44 | 2,22 | 1,44 | 2 | 20 | 4,50 | 4,50 | 4,50 | 2,50 | 3,00 | 2 | 0 | 0 |
| 50 | 1 | 43 | 3,30 | 3,93 | 3,15 | 4,33 | 3,44 | 2,53 | 11 | 19 | 3,00 | 4,50 | 4,50 | 2,50 | 3,00 | 2 | 1 | 3 |
| 51 | 2 | 29 | 3,70 | 2,87 | 3,15 | 3,00 | 3,00 | 3,11 | 6 | 17 | 3,00 | 3,50 | 2,50 | 1,50 | 5,00 | 4 | 0 | 0 |
| 52 | 1 | 42 | 2,50 | 2,73 | 3,23 | 3,33 | 2,33 | 2,13 | 1 | 17 | 3,00 | 4,00 | 3,00 | 4,00 | 3,00 | 4 | 0 | 0 |
| 53 | 2 | 49 | 3,50 | 3,27 | 3,85 | 3,78 | 3,22 | 2,54 | 8 | 20 | 2,00 | 4,50 | 5,00 | 3,00 | 4,00 | 4 | 1 | 2 |
| 54 | 2 | 47 | 2,40 | 3,00 | 2,69 | 4,33 | 1,67 | 1,68 | 3 | 15 | 4,00 | 4,00 | 3,00 | 4,00 | 3,50 | 3 | 0 | 0 |
| 55 | 2 | 57 | 2,90 | 3,13 | 3,54 | 3,22 | 2,78 | 2,29 | 14 | 20 | 3,50 | 3,00 | 2,50 | 3,50 | 2,50 | 2 | 0 | 0 |
| 56 | 1 | 55 | 4,20 | 4,20 | 4,23 | 3,00 | 3,44 | 3,40 | 14 | 11 | 3,00 | 4,50 | 2,50 | 3,00 | 2,50 | 3 | 0 | 0 |
| 57 | 1 | 30 | 3,30 | 3,13 | 2,77 | 3,22 | 2,44 | 2,59 | 6 | 13 | 3,00 | 4,00 | 3,50 | 3,00 | 3,00 | 4 | 0 | 0 |
| 58 | 2 | 56 | 4,50 | 3,60 | 3,69 | 2,67 | 4,33 | 4,30 | 12 | 14 | 3,00 | 5,00 | 2,00 | 3,50 | 3,00 | 3 | 1 | 2 |
| 59 | 1 | 56 | 3,90 | 3,47 | 3,31 | 4,22 | 3,00 | 2,62 | 2 | 17 | 4,50 | 4,00 | 3,50 | 3,00 | 3,50 | 3 | 1 | 2 |
| 60 | 2 | 52 | 3,60 | 2,53 | 3,00 | 2,89 | 2,11 | 2,98 | 4 | 19 | 5,00 | 2,00 | 2,50 | 3,00 | 3,50 | 3 | 0 | 0 |
| 61 | 2 | 50 | 3,90 | 3,47 | 3,85 | 2,78 | 3,44 | 3,33 | 7 | 18 | 3,00 | 4,50 | 2,50 | 2,00 | 4,50 | 3 | 0 | 0 |
| 62 | 1 | 23 | 3,40 | 3,40 | 2,46 | 4,00 | 2,56 | 2,49 | 7 | 12 | 2,50 | 3,00 | 1,50 | 4,00 | 4,00 | 3 | 1 | 2 |
| 63 | 2 | 26 | 4,30 | 4,07 | 3,69 | 3,44 | 3,56 | 3,35 | 13 | 15 | 2,50 | 3,50 | 2,50 | 3,00 | 5,00 | 4 | 0 | 0 |
| 64 | 1 | 22 | 4,10 | 3,67 | 3,69 | 3,67 | 3,56 | 3,14 | 13 | 13 | 3,50 | 3,50 | 2,00 | 2,00 | 4,50 | 3 | 0 | 0 |
| 65 | 2 | 34 | 2,90 | 2,93 | 3,08 | 4,00 | 2,11 | 1,85 | 6 | 16 | 2,50 | 2,50 | 3,50 | 4,00 | 2,50 | 3 | 0 | 0 |

| | | | | | | | | | | | | | | | | | | | |
|---|---|---|---|---|---|---|---|---|---|---|---|---|---|---|---|---|---|---|---|
| 66 | 2 | 20 | 2,00 | 3,20 | 3,15 | 2,89 | 2,89 | 2,46 | 7 | 13 | 2,00 | 2,50 | 3,00 | 3,00 | 3,00 | 3 | 0 | 0 |
| 67 | 2 | 33 | 1,30 | 2,73 | 2,77 | 3,00 | 3,00 | 2,85 | 8 | 16 | 3,50 | 3,50 | 4,50 | 3,50 | 1,00 | 2 | 1 | 1 |
| 68 | 2 | 53 | 3,50 | 2,53 | 3,31 | 2,22 | 2,44 | 3,36 | 2 | 20 | 4,50 | 4,50 | 2,50 | 2,50 | 4,00 | 3 | 0 | 0 |
| 69 | 2 | 33 | 2,20 | 2,40 | 3,38 | 3,89 | 3,00 | 2,33 | 2 | 20 | 2,50 | 5,00 | 3,50 | 1,50 | 2,00 | 4 | 0 | 0 |
| 70 | 1 | 24 | 3,00 | 1,87 | 3,38 | 3,56 | 2,44 | 2,76 | 4 | 18 | 3,00 | 4,50 | 2,50 | 3,50 | 5,00 | 4 | 0 | 0 |
| 71 | 1 | 37 | 4,80 | 3,00 | 3,54 | 3,67 | 2,78 | 3,41 | 8 | 22 | 3,50 | 5,00 | 3,00 | 3,00 | 2,00 | 2 | 0 | 0 |
| 72 | 1 | 24 | 2,30 | 4,53 | 2,15 | 2,89 | 3,44 | 3,26 | 9 | 17 | 4,50 | 4,50 | 4,00 | 5,00 | 3,00 | 3 | 1 | 2 |
| 73 | 1 | 35 | 2,40 | 3,20 | 3,38 | 4,56 | 2,78 | 1,64 | 2 | 12 | 3,00 | 2,00 | 3,00 | 2,50 | 3,00 | 3 | 0 | 0 |
| 74 | 2 | 25 | 2,60 | 3,13 | 3,69 | 4,44 | 2,33 | 1,35 | 11 | 17 | 5,00 | 3,50 | 2,00 | 3,00 | 4,00 | 3 | 1 | 2 |
| 75 | 2 | 22 | 3,10 | 3,20 | 3,23 | 3,67 | 3,11 | 2,42 | 7 | 12 | 4,00 | 2,50 | 2,50 | 5,00 | 2,50 | 3 | 1 | 2 |
| 76 | 1 | 50 | 2,60 | 3,73 | 3,31 | 3,78 | 2,89 | 1,88 | 8 | 19 | 3,00 | 3,50 | 3,50 | 3,50 | 2,50 | 2 | 1 | 1 |
| 77 | 2 | 24 | 2,30 | 3,13 | 2,85 | 2,67 | 2,22 | 2,51 | 8 | 12 | 1,00 | 3,50 | 2,50 | 1,50 | 2,50 | 4 | 0 | 0 |
| 78 | 2 | 38 | 2,90 | 3,60 | 3,31 | 4,44 | 3,78 | 2,59 | 13 | 17 | 2,50 | 3,50 | 1,50 | 2,00 | 4,00 | 2 | 0 | 0 |
| 79 | 1 | 33 | 3,20 | 4,13 | 3,69 | 4,56 | 4,00 | 2,82 | 2 | 18 | 3,50 | 3,50 | 2,00 | 2,50 | 1,50 | 3 | 1 | 2 |
| 80 | 2 | 32 | 2,70 | 3,93 | 3,69 | 4,22 | 1,67 | 0,91 | 1 | 20 | 4,00 | 5,00 | 3,00 | 4,00 | 4,00 | 3 | 0 | 0 |
| 81 | 1 | 24 | 4,10 | 3,93 | 2,92 | 2,33 | 1,56 | 3,31 | 5 | 17 | 3,50 | 2,50 | 2,50 | 4,50 | 3,00 | 3 | 0 | 0 |
| 82 | 2 | 24 | 4,00 | 3,53 | 3,15 | 3,44 | 2,67 | 2,78 | 3 | 19 | 4,00 | 3,50 | 2,50 | 3,00 | 3,50 | 3 | 0 | 0 |
| 83 | 2 | 52 | 3,00 | 3,47 | 3,08 | 3,33 | 2,33 | 2,11 | 5 | 21 | 3,00 | 4,00 | 2,50 | 4,00 | 3,00 | 2 | 0 | 0 |
| 84 | 1 | 36 | 2,60 | 3,20 | 3,15 | 3,33 | 2,33 | 1,98 | 10 | 21 | 4,00 | 3,00 | 3,50 | 3,00 | 4,00 | 2 | 1 | 1 |
| 85 | 2 | 20 | 2,80 | 3,33 | 2,77 | 3,00 | 4,44 | 3,71 | 5 | 12 | 3,50 | 5,00 | 2,50 | 5,00 | 3,00 | 3 | 0 | 0 |
| 86 | 1 | 62 | 3,40 | 4,13 | 3,92 | 4,44 | 3,67 | 2,62 | 15 | 16 | 3,50 | 3,00 | 3,00 | 4,00 | 2,50 | 2 | 0 | 0 |
| 87 | 2 | 25 | 2,80 | 3,47 | 3,15 | 4,56 | 2,67 | 1,73 | 5 | 19 | 3,50 | 4,00 | 2,50 | 3,50 | 2,50 | 4 | 0 | 0 |
| 88 | 2 | 24 | 3,30 | 3,20 | 3,92 | 3,56 | 3,11 | 2,45 | 15 | 14 | 4,00 | 2,50 | 1,50 | 3,00 | 4,00 | 3 | 1 | 2 |
| 89 | 2 | 22 | 2,80 | 2,53 | 4,08 | 2,78 | 2,33 | 2,57 | 6 | 15 | 3,00 | 3,50 | 2,00 | 2,00 | 3,50 | 3 | 0 | 0 |
| 90 | 1 | 20 | 2,60 | 3,87 | 2,46 | 3,67 | 2,22 | 2,05 | 4 | 13 | 3,50 | 5,00 | 2,50 | 4,50 | 5,00 | 3 | 0 | 0 |
| 91 | 1 | 35 | 3,40 | 3,87 | 3,38 | 3,67 | 2,56 | 2,11 | 9 | 8 | 2,00 | 2,50 | 1,50 | 4,00 | 3,00 | 3 | 1 | 2 |
| 92 | 2 | 21 | 4,10 | 1,73 | 4,69 | 2,89 | 2,67 | 3,75 | 15 | 9 | 2,50 | 2,50 | 3,00 | 2,00 | 4,00 | 3 | 0 | 0 |
| 93 | 2 | 28 | 4,40 | 3,27 | 3,46 | 3,67 | 2,56 | 2,95 | 13 | 14 | 2,00 | 2,00 | 2,50 | 1,00 | 4,50 | 3 | 0 | 0 |
| 94 | 2 | 29 | 3,00 | 3,73 | 2,77 | 4,11 | 2,33 | 1,89 | 13 | 17 | 2,50 | 2,50 | 1,50 | 3,50 | 3,00 | 3 | 0 | 0 |
| 95 | 2 | 28 | 4,00 | 2,93 | 3,00 | 3,00 | 3,11 | 3,36 | 14 | 12 | 4,50 | 3,00 | 2,00 | 2,50 | 3,50 | 3 | 0 | 0 |
| 96 | 1 | 23 | 3,30 | 3,67 | 3,38 | 3,67 | 2,67 | 2,12 | 2 | 12 | 3,50 | 3,50 | 3,00 | 4,50 | 4,50 | 4 | 0 | 0 |
| 97 | 1 | 57 | 2,90 | 3,73 | 3,69 | 3,44 | 2,89 | 2,07 | 13 | 16 | 3,00 | 3,50 | 2,00 | 3,00 | 3,00 | 3 | 1 | 1 |
| 98 | 2 | 54 | 3,20 | 2,73 | 3,69 | 3,78 | 2,89 | 2,37 | 16 | 16 | 4,00 | 3,50 | 1,50 | 4,00 | 4,00 | 3 | 0 | 0 |

| | | | | | | | | | | | | | | | | | | | |
|---|---|---|---|---|---|---|---|---|---|---|---|---|---|---|---|---|---|---|---|
| 99 | 1 | 50 | 3,70 | 3,33 | 3,92 | 3,78 | 2,78 | 2,39 | 15 | 20 | 5,00 | 5,00 | 1,00 | 3,00 | 2,50 | 4 | 0 | 0 |
| ## | 2 | 39 | 4,20 | 2,80 | 3,54 | 3,56 | 2,56 | 2,95 | 4 | 19 | 1,50 | 5,00 | 3,00 | 2,00 | 3,50 | 4 | 0 | 0 |
| ## | 2 | 26 | 3,80 | 3,40 | 3,54 | 3,11 | 2,44 | 2,64 | 4 | 14 | 3,00 | 3,00 | 3,50 | 3,00 | 4,00 | 4 | 0 | 0 |
| ## | 2 | 38 | 2,90 | 2,73 | 3,23 | 3,89 | 2,67 | 2,17 | 14 | 18 | 4,00 | 5,00 | 4,50 | 3,00 | 2,00 | 3 | 1 | 2 |
| ## | 1 | 46 | 3,60 | 2,93 | 3,54 | 4,33 | 2,44 | 2,20 | 2 | 22 | 4,00 | 5,00 | 3,00 | 3,00 | 4,50 | 4 | 0 | 0 |
| ## | 2 | 28 | 2,60 | 3,47 | 2,69 | 3,33 | 2,44 | 2,19 | 8 | 13 | 3,50 | 3,00 | 3,00 | 2,00 | 4,00 | 2 | 0 | 0 |
| ## | 1 | 31 | 2,80 | 3,07 | 2,54 | 3,56 | 2,11 | 2,24 | 7 | 18 | 4,50 | 4,00 | 3,00 | 3,50 | 2,00 | 3 | 1 | 1 |
| ## | 1 | 31 | 2,80 | 3,27 | 3,31 | 4,44 | 2,11 | 1,41 | 10 | 16 | 3,00 | 3,50 | 3,50 | 3,50 | 2,50 | 3 | 0 | 0 |
| ## | 2 | 48 | 3,50 | 3,60 | 3,69 | 3,89 | 3,22 | 2,46 | 3 | 19 | 3,50 | 3,50 | 3,50 | 3,00 | 3,50 | 3 | 0 | 0 |
| ## | 1 | 31 | 2,40 | 3,00 | 3,31 | 4,22 | 2,33 | 1,53 | 5 | 17 | 3,50 | 5,00 | 2,50 | 4,50 | 3,00 | 3 | 0 | 0 |
| ## | 2 | 27 | 3,50 | 3,33 | 2,77 | 4,00 | 3,78 | 3,13 | 10 | 17 | 2,50 | 5,00 | 2,00 | 3,00 | 5,00 | 3 | 1 | 1 |
| ## | 2 | 52 | 3,10 | 3,87 | 3,23 | 3,22 | 2,78 | 2,33 | 19 | 18 | 3,50 | 5,00 | 4,50 | 3,00 | 5,00 | 3 | 0 | 0 |
| ## | 1 | 52 | 3,80 | 3,07 | 3,23 | 3,44 | 3,00 | 2,88 | 12 | 18 | 3,00 | 3,00 | 2,00 | 3,50 | 4,50 | 3 | 0 | 0 |
| ## | 1 | 36 | 1,60 | 1,93 | 4,38 | 4,22 | 1,22 | 2,09 | 0 | 16 | 4,00 | 4,50 | 5,00 | 3,50 | 1,50 | 3 | 0 | 0 |
| ## | 2 | 27 | 2,30 | 3,33 | 3,54 | 4,33 | 2,78 | 1,54 | 5 | 19 | 3,50 | 4,50 | 3,50 | 5,00 | 1,00 | 3 | 0 | 0 |
| ## | 2 | 28 | 3,10 | 3,07 | 3,31 | 4,11 | 3,00 | 2,23 | 7 | 15 | 3,50 | 4,50 | 4,00 | 3,00 | 3,00 | 3 | 1 | 2 |
| ## | 1 | 50 | 3,40 | 3,07 | 3,77 | 3,89 | 2,89 | 2,30 | 15 | 13 | 3,50 | 4,50 | 2,50 | 3,00 | 2,00 | 3 | 0 | 0 |
| ## | 2 | 26 | 3,40 | 2,60 | 2,77 | 4,11 | 2,33 | 2,50 | 4 | 9 | 3,50 | 5,00 | 1,00 | 5,00 | 3,00 | 4 | 1 | 2 |
| ## | 2 | 43 | 2,90 | 3,33 | 3,69 | 4,11 | 3,22 | 2,13 | 6 | 19 | 3,50 | 3,00 | 4,00 | 3,00 | 3,50 | 2 | 0 | 0 |
| ## | 2 | 29 | 2,30 | 3,20 | 3,46 | 3,33 | 2,89 | 2,11 | 6 | 15 | 3,00 | 3,00 | 3,00 | 3,00 | 2,50 | 3 | 1 | 1 |
| ## | 2 | 29 | 3,30 | 4,20 | 3,92 | 4,00 | 3,89 | 2,83 | 7 | 16 | 2,00 | 4,00 | 3,00 | 4,00 | 4,00 | 3 | 1 | 2 |
| ## | 1 | 27 | 2,70 | 2,93 | 3,62 | 3,89 | 2,11 | 1,59 | 7 | 14 | 4,00 | 3,00 | 3,00 | 3,00 | 4,50 | 4 | 0 | 0 |
| ## | 2 | 58 | 3,30 | 3,00 | 2,62 | 3,33 | 3,00 | 2,90 | 7 | 17 | 3,50 | 4,00 | 3,50 | 2,50 | 3,50 | 3 | 0 | 0 |
| ## | 1 | 28 | 1,90 | 2,93 | 3,54 | 3,67 | 2,00 | 1,51 | 1 | 17 | 3,00 | 4,00 | 3,00 | 3,50 | 3,50 | 3 | 0 | 0 |
| ## | 1 | 21 | 2,00 | 3,00 | 3,23 | 4,44 | 1,89 | 1,26 | 4 | 17 | 3,00 | 4,50 | 3,00 | 3,50 | 3,00 | 3 | 0 | 0 |
| ## | 2 | 41 | 2,50 | 3,13 | 3,38 | 3,78 | 2,22 | 1,57 | 3 | 17 | 4,00 | 4,00 | 3,50 | 4,00 | 2,50 | 0 | 0 | 0 |
| ## | 1 | 26 | 3,50 | 3,40 | 4,00 | 3,78 | 2,89 | 2,29 | 12 | 15 | 2,00 | 3,50 | 2,00 | 2,50 | 3,00 | 3 | 0 | 0 |
| ## | 2 | 29 | 2,50 | 3,47 | 3,54 | 4,33 | 3,33 | 2,03 | 6 | 17 | 3,50 | 4,00 | 4,50 | 4,50 | 2,00 | 3 | 0 | 0 |
| ## | 1 | 25 | 2,30 | 3,20 | 4,23 | 4,00 | 2,22 | 1,24 | 6 | 16 | 3,50 | 5,00 | 2,00 | 3,00 | 3,50 | 4 | 0 | 0 |
| ## | 1 | 52 | 3,00 | 2,67 | 3,54 | 3,67 | 3,22 | 2,58 | 8 | 12 | 4,00 | 2,00 | 2,00 | 1,50 | 3,00 | 2 | 0 | 0 |
| ## | 2 | 32 | 3,10 | 2,87 | 3,46 | 4,22 | 2,67 | 2,05 | 8 | 21 | 1,00 | 5,00 | 4,00 | 1,50 | 2,00 | 3 | 0 | 0 |
| ## | 1 | 41 | 3,80 | 2,73 | 3,15 | 3,44 | 1,67 | 2,62 | 3 | 16 | 4,00 | 4,00 | 2,50 | 2,50 | 3,00 | 4 | 0 | 0 |
| ## | 2 | 59 | 3,50 | 3,47 | 3,31 | 2,67 | 3,00 | 3,01 | 6 | 18 | 3,00 | 2,50 | 1,50 | 3,00 | 3,00 | 4 | 0 | 0 |

| | | | | | | | | | | | | | | | | | | |
|---|---|---|---|---|---|---|---|---|---|---|---|---|---|---|---|---|---|---|
| ## | 2 | 40 | 2,80 | 3,13 | 4,38 | 4,44 | 1,56 | 1,22 | 1 | 20 | 3,50 | 4,00 | 2,50 | 4,00 | 4,00 | 3 | 0 | 0 |
| ## | 2 | 45 | 2,00 | 2,87 | 2,92 | 3,78 | 2,11 | 1,81 | 4 | 19 | 4,00 | 5,00 | 3,00 | 3,00 | 5,00 | 2 | 0 | 0 |
| ## | 2 | 40 | 2,60 | 2,47 | 2,85 | 3,89 | 3,00 | 2,56 | 2 | 20 | 3,50 | 3,50 | 2,00 | 3,00 | 4,50 | 4 | 0 | 0 |
| ## | 1 | 40 | 3,80 | 3,20 | 3,23 | 3,33 | 3,00 | 2,89 | 10 | 19 | 1,00 | 3,50 | 4,00 | 3,00 | 3,50 | 3 | 0 | 0 |
| ## | 2 | 25 | 2,30 | 2,47 | 3,54 | 3,22 | 1,44 | 2,07 | 17 | 12 | 3,50 | 3,50 | 3,00 | 4,00 | 2,50 | 3 | 1 | 1 |
| ## | 2 | 33 | 2,70 | 3,00 | 3,77 | 3,78 | 2,44 | 1,73 | 3 | 20 | 4,00 | 4,50 | 2,50 | 3,50 | 3,00 | 3 | 0 | 0 |
| ## | 1 | 49 | 3,30 | 3,80 | 3,23 | 3,89 | 3,11 | 2,35 | 12 | 17 | 4,50 | 3,50 | 4,50 | 2,50 | 3,50 | 3 | 0 | 0 |
| ## | 1 | 62 | 2,50 | 2,47 | 4,31 | 3,78 | 2,22 | 1,91 | 4 | 19 | 4,50 | 5,00 | 4,00 | 2,00 | 1,00 | 2 | 0 | 0 |
| ## | 1 | 32 | 2,50 | 2,53 | 3,85 | 4,11 | 1,78 | 1,58 | 6 | 18 | 3,50 | 5,00 | 3,00 | 3,50 | 5,00 | 3 | 0 | 0 |
| ## | 2 | 62 | 3,10 | 3,00 | 4,23 | 3,56 | 2,11 | 1,91 | 1 | 19 | 3,00 | 4,00 | 3,50 | 3,50 | 3,50 | 2 | 0 | 0 |
| ## | 1 | 70 | 2,60 | 2,87 | 3,31 | 4,44 | 2,33 | 1,64 | 8 | 21 | 2,00 | 3,50 | 2,50 | 4,00 | 3,00 | 1 | 0 | 0 |
| ## | 2 | 74 | 2,40 | 2,07 | 3,77 | 4,67 | 2,00 | 1,97 | 8 | 20 | 3,50 | 4,00 | 3,50 | 2,00 | 2,50 | 1 | 0 | 0 |
| ## | 2 | 22 | 2,50 | 3,93 | 3,31 | 4,44 | 2,78 | 1,56 | 7 | 15 | 3,50 | 4,00 | 2,00 | 3,00 | 2,00 | 3 | 0 | 0 |
| ## | 2 | 56 | 2,20 | 2,47 | 4,15 | 4,00 | 2,44 | 1,84 | 5 | 20 | 3,50 | 5,00 | 4,00 | 4,00 | 1,00 | 2 | 0 | 0 |
| ## | 2 | 31 | 1,40 | 3,27 | 3,31 | 3,78 | 2,11 | 1,49 | 5 | 17 | 4,00 | 4,00 | 4,50 | 3,50 | 3,00 | 3 | 0 | 0 |
| ## | 2 | 25 | 2,80 | 3,33 | 3,15 | 4,22 | 3,00 | 2,04 | 7 | 14 | 3,00 | 4,00 | 2,50 | 2,50 | 4,00 | 4 | 1 | 2 |
| ## | 1 | 25 | 3,10 | 2,93 | 3,85 | 2,56 | 2,56 | 2,75 | 8 | 19 | 1,50 | 4,00 | 2,50 | 2,00 | 3,50 | 4 | 0 | 0 |
| ## | 2 | 24 | 2,50 | 2,47 | 3,92 | 3,44 | 2,22 | 2,05 | 9 | 13 | 5,00 | 2,00 | 4,00 | 2,00 | 1,00 | 3 | 0 | 0 |
| ## | 1 | 50 | 2,90 | 3,07 | 4,00 | 3,67 | 1,78 | 1,60 | 3 | 17 | 3,50 | 4,00 | 2,50 | 2,50 | 3,00 | 4 | 0 | 0 |
| ## | 2 | 21 | 3,20 | 3,73 | 2,69 | 4,00 | 2,67 | 2,24 | 9 | 11 | 3,50 | 2,50 | 1,50 | 2,00 | 4,00 | 2 | 0 | 0 |
| ## | 2 | 20 | 2,60 | 3,47 | 3,46 | 4,00 | 3,00 | 1,87 | 9 | 19 | 3,00 | 3,00 | 2,50 | 3,00 | 4,50 | 3 | 0 | 0 |
| ## | 2 | 23 | 2,60 | 3,00 | 3,69 | 3,44 | 1,78 | 1,66 | 8 | 17 | 3,00 | 3,00 | 3,00 | 3,00 | 4,00 | 3 | 0 | 0 |
| ## | 2 | 71 | 3,50 | 2,40 | 4,08 | 4,56 | 3,78 | 3,14 | 7 | 17 | 2,00 | 2,50 | 2,50 | 4,00 | 2,50 | 1 | 0 | 0 |
| ## | 1 | 27 | 1,90 | 3,13 | 3,69 | 4,44 | 1,78 | 0,89 | 3 | 19 | 3,50 | 5,00 | 2,50 | 3,50 | 3,50 | 3 | 0 | 0 |
| ## | 2 | 24 | 2,10 | 2,53 | 3,31 | 4,00 | 2,11 | 1,76 | 3 | 19 | 3,50 | 3,50 | 2,50 | 3,50 | 3,50 | 4 | 0 | 0 |
| ## | 2 | 31 | 2,70 | 3,73 | 3,62 | 3,78 | 2,67 | 1,67 | 11 | 10 | 2,50 | 5,00 | 1,00 | 4,00 | 2,50 | 3 | 0 | 0 |
| ## | 1 | 33 | 2,80 | 3,27 | 3,31 | 3,00 | 2,67 | 2,35 | 12 | 17 | 2,50 | 2,00 | 4,00 | 4,50 | 1,50 | 2 | 1 | 1 |
| ## | 1 | 31 | 2,50 | 2,67 | 2,92 | 3,00 | 2,78 | 2,68 | 14 | 14 | 2,50 | 2,50 | 2,50 | 4,50 | 3,00 | 3 | 0 | 0 |
| ## | 1 | 51 | 3,30 | 2,73 | 3,23 | 4,00 | 3,33 | 2,74 | 8 | 14 | 3,00 | 3,50 | 2,00 | 4,00 | 3,50 | 3 | 0 | 0 |
| ## | 2 | 23 | 2,70 | 2,87 | 3,92 | 4,11 | 2,56 | 1,73 | 7 | 15 | 3,50 | 4,00 | 3,50 | 3,50 | 2,50 | 4 | 0 | 0 |
| ## | 2 | 36 | 3,40 | 3,67 | 2,69 | 3,11 | 3,89 | 3,43 | 8 | 16 | 3,00 | 3,50 | 2,50 | 4,50 | 2,50 | 4 | 1 | 2 |
| ## | 2 | 56 | 2,50 | 3,20 | 3,46 | 3,89 | 3,00 | 1,96 | 3 | 20 | 3,00 | 4,00 | 3,50 | 4,00 | 3,50 | 4 | 0 | 0 |
| ## | 2 | 40 | 4,90 | 4,27 | 3,77 | 3,56 | 4,22 | 4,17 | 3 | 16 | 3,00 | 2,50 | 2,50 | 3,50 | 3,00 | 3 | 1 | 2 |

| | | | | | | | | | | | | | | | | | | |
|---|---|---|---|---|---|---|---|---|---|---|---|---|---|---|---|---|---|---|
| ## | 2 | 36 | 2,20 | 3,20 | 3,38 | 3,89 | 2,33 | 1,46 | 6 | 20 | 3,00 | 3,00 | 1,50 | 4,00 | 1,00 | 3 | 0 | 0 |
| ## | 1 | 36 | 3,80 | 3,87 | 3,46 | 4,11 | 2,78 | 2,36 | 8 | 19 | 3,50 | 2,00 | 4,00 | 4,00 | 4,00 | 4 | 0 | 0 |
| ## | 2 | 39 | 2,40 | 3,53 | 3,69 | 4,00 | 2,33 | 1,22 | 2 | 18 | 4,50 | 2,50 | 3,00 | 3,50 | 4,50 | 2 | 1 | 2 |
| ## | 1 | 34 | 3,00 | 3,33 | 3,38 | 3,67 | 2,78 | 2,08 | 6 | 20 | 4,00 | 5,00 | 2,00 | 2,00 | 2,50 | 3 | 0 | 0 |
| ## | 1 | 37 | 1,50 | 2,67 | 3,69 | 4,44 | 3,00 | 2,02 | 5 | 17 | 4,50 | 4,00 | 3,00 | 4,00 | 1,50 | 3 | 0 | 0 |
| ## | 1 | 36 | 3,20 | 3,40 | 3,46 | 3,67 | 3,44 | 2,60 | 7 | 16 | 2,00 | 5,00 | 3,00 | 5,00 | 4,00 | 3 | 0 | 0 |
| ## | 2 | 36 | 3,80 | 2,93 | 3,46 | 3,56 | 2,78 | 2,71 | 7 | 20 | 3,00 | 4,00 | 1,00 | 3,00 | 5,00 | 4 | 0 | 0 |
| ## | 2 | 60 | 4,20 | 2,07 | 3,31 | 4,11 | 3,44 | 3,59 | 17 | 19 | 2,00 | 4,50 | 3,00 | 1,50 | 5,00 | 4 | 0 | 0 |
| ## | 1 | 43 | 2,40 | 3,00 | 3,15 | 3,44 | 2,33 | 1,94 | 3 | 14 | 3,00 | 3,50 | 3,50 | 3,50 | 3,50 | 3 | 1 | 2 |
| ## | 1 | 47 | 2,60 | 2,67 | 3,62 | 3,67 | 2,56 | 2,02 | 6 | 15 | 4,50 | 3,00 | 2,00 | 2,00 | 3,50 | 2 | 0 | 0 |
| ## | 2 | 58 | 2,70 | 2,93 | 3,08 | 4,11 | 2,11 | 1,72 | 4 | 15 | 2,50 | 3,00 | 1,50 | 2,00 | 3,50 | 2 | 0 | 0 |
| ## | 1 | 53 | 2,20 | 3,87 | 2,62 | 3,00 | 2,44 | 2,33 | 14 | 14 | 2,00 | 4,00 | 3,00 | 3,50 | 1,50 | 2 | 0 | 0 |
| ## | 1 | 56 | 1,80 | 3,00 | 3,31 | 3,33 | 3,00 | 2,27 | 5 | 12 | 3,00 | 3,50 | 3,50 | 2,50 | 2,00 | 3 | 1 | 1 |
| ## | 1 | 63 | 2,50 | 3,60 | 2,92 | 2,22 | 2,67 | 2,93 | 12 | 20 | 3,00 | 4,50 | 3,00 | 2,50 | 3,00 | 2 | 1 | 1 |
| ## | 1 | 48 | 2,50 | 2,80 | 3,92 | 4,11 | 2,11 | 1,46 | 5 | 16 | 3,00 | 4,50 | 3,00 | 3,00 | 2,00 | 3 | 0 | 0 |
| ## | 1 | 54 | 2,40 | 2,93 | 2,46 | 3,44 | 2,56 | 2,44 | 2 | 14 | 4,00 | 4,50 | 3,50 | 2,00 | 3,50 | 3 | 0 | 0 |
| ## | 1 | 56 | 2,70 | 3,07 | 3,46 | 3,78 | 2,89 | 2,04 | 4 | 14 | 3,00 | 3,50 | 3,50 | 4,50 | 1,50 | 1 | 0 | 0 |
| ## | 1 | 57 | 1,50 | 2,60 | 3,46 | 3,33 | 2,00 | 2,01 | 0 | 21 | 3,00 | 5,00 | 4,00 | 2,50 | 3,50 | 3 | 0 | 0 |
| ## | 2 | 55 | 4,60 | 2,93 | 3,92 | 3,56 | 3,00 | 3,36 | 22 | 18 | 3,00 | 3,00 | 3,00 | 5,00 | 4,00 | 3 | 0 | 0 |
| ## | 2 | 47 | 2,20 | 2,80 | 2,92 | 4,00 | 1,89 | 1,72 | 9 | 16 | 2,00 | 3,50 | 3,00 | 4,00 | 3,00 | 3 | 0 | 0 |
| ## | 2 | 35 | 3,10 | 3,33 | 2,62 | 3,22 | 1,89 | 2,37 | 8 | 17 | 3,00 | 4,50 | 4,00 | 4,00 | 2,00 | 3 | 1 | 2 |
| ## | 2 | 43 | 3,70 | 3,60 | 2,85 | 2,78 | 4,33 | 3,98 | 10 | 13 | 2,50 | 3,50 | 2,00 | 2,00 | 3,50 | 3 | 0 | 0 |
| ## | 1 | 25 | 3,30 | 3,20 | 2,85 | 3,67 | 2,78 | 2,48 | 8 | 17 | 3,00 | 4,50 | 4,00 | 4,00 | 2,00 | 4 | 0 | 0 |
| ## | 1 | 62 | 2,40 | 3,20 | 3,08 | 3,78 | 2,67 | 1,89 | 5 | 13 | 2,00 | 4,50 | 3,00 | 3,50 | 3,00 | 3 | 1 | 1 |
| ## | 1 | 51 | 3,50 | 3,53 | 2,92 | 3,11 | 2,56 | 2,65 | 5 | 18 | 3,00 | 4,00 | 2,50 | 3,00 | 3,00 | 2 | 0 | 0 |
| ## | 1 | 56 | 3,00 | 2,93 | 2,85 | 2,78 | 3,11 | 3,05 | 16 | 13 | 3,00 | 3,50 | 4,00 | 4,50 | 2,50 | 3 | 1 | 2 |
| ## | 1 | 26 | 3,30 | 2,67 | 2,77 | 2,44 | 3,11 | 3,48 | 13 | 17 | 3,00 | 3,50 | 4,00 | 3,50 | 3,00 | 3 | 1 | 2 |
| ## | 1 | 34 | 3,30 | 3,27 | 3,46 | 3,00 | 3,67 | 3,13 | 9 | 19 | 4,50 | 4,50 | 5,00 | 2,00 | 2,00 | 4 | 1 | 2 |
| ## | 2 | 44 | 2,10 | 3,20 | 3,23 | 3,78 | 2,11 | 1,47 | 10 | 17 | 2,50 | 3,50 | 3,00 | 3,00 | 3,50 | 4 | 1 | 2 |
| ## | 1 | 45 | 3,30 | 3,20 | 3,15 | 3,11 | 3,67 | 3,15 | 9 | 18 | 4,50 | 3,00 | 5,00 | 2,00 | 2,00 | 4 | 1 | 2 |
| ## | 1 | 37 | 4,80 | 4,40 | 3,46 | 3,89 | 4,11 | 4,00 | 1 | 14 | 2,00 | 4,50 | 2,50 | 5,00 | 5,00 | 3 | 0 | 0 |
| ## | 2 | 32 | 3,10 | 2,87 | 4,08 | 3,22 | 3,00 | 2,56 | 7 | 17 | 2,50 | 4,00 | 2,50 | 3,50 | 5,00 | 3 | 0 | 0 |
| ## | 2 | 36 | 3,20 | 2,80 | 3,54 | 3,00 | 3,00 | 2,79 | 1 | 20 | 3,00 | 4,50 | 2,50 | 3,00 | 4,00 | 4 | 0 | 0 |

| | | | | | | | | | | | | | | | | | | | |
|---|---|---|---|---|---|---|---|---|---|---|---|---|---|---|---|---|---|---|---|
| ## | 2 | 47 | 3,60 | 3,53 | 3,46 | 3,11 | 1,89 | 2,35 | 10 | 18 | 3,00 | 2,50 | 3,50 | 1,50 | 3,00 | 3 | 0 | 0 |
| ## | 1 | 37 | 2,20 | 4,27 | 3,31 | 4,56 | 2,78 | 1,52 | 7 | 8 | 3,50 | 3,50 | 2,50 | 2,50 | 3,50 | 3 | 0 | 0 |
| ## | 1 | 43 | 2,60 | 2,67 | 3,00 | 4,00 | 2,44 | 2,05 | 2 | 14 | 3,50 | 4,00 | 4,00 | 3,50 | 2,50 | 3 | 0 | 0 |
| ## | 1 | 70 | 2,00 | 2,13 | 3,00 | 2,89 | 2,00 | 2,70 | 11 | 21 | 3,00 | 5,00 | 3,00 | 3,50 | 3,00 | 2 | 0 | 0 |
| ## | 2 | 65 | 2,40 | 2,53 | 3,54 | 3,56 | 2,00 | 1,90 | 9 | 21 | 4,00 | 3,00 | 3,00 | 3,50 | 3,50 | 2 | 0 | 0 |
| ## | 2 | 23 | 3,00 | 3,53 | 3,62 | 2,44 | 2,56 | 2,67 | 0 | 14 | 3,50 | 4,00 | 2,50 | 3,00 | 4,00 | 3 | 0 | 0 |
| ## | 2 | 45 | 3,20 | 3,00 | 3,54 | 3,56 | 2,89 | 2,37 | 11 | 16 | 3,00 | 4,50 | 3,50 | 4,00 | 1,50 | 2 | 0 | 0 |
| ## | 2 | 70 | 1,60 | 2,13 | 3,46 | 3,56 | 2,11 | 2,24 | 13 | 15 | 4,00 | 4,00 | 3,50 | 5,00 | 2,00 | 2 | 0 | 0 |
| ## | 1 | 48 | 2,80 | 3,33 | 2,46 | 3,56 | 3,33 | 2,80 | 4 | 16 | 4,00 | 3,00 | 5,00 | 2,50 | 1,50 | 2 | 0 | 0 |
| ## | 1 | 49 | 3,70 | 3,47 | 3,38 | 4,00 | 3,78 | 3,03 | 4 | 19 | 4,50 | 5,00 | 3,50 | 3,00 | 2,50 | 2 | 1 | 2 |
| ## | 2 | 28 | 3,40 | 2,80 | 3,69 | 3,56 | 2,33 | 2,28 | 7 | 16 | 4,00 | 3,50 | 1,50 | 2,00 | 2,00 | 3 | 1 | 2 |
| ## | 2 | 23 | 3,40 | 4,13 | 3,62 | 2,89 | 2,44 | 2,47 | 3 | 12 | 4,50 | 4,00 | 2,50 | 3,50 | 3,50 | 4 | 0 | 0 |
| ## | 2 | 23 | 2,50 | 4,07 | 3,00 | 2,89 | 2,56 | 2,32 | 6 | 12 | 4,00 | 3,00 | 2,50 | 4,00 | 2,50 | 4 | 0 | 0 |
| ## | 1 | 21 | 3,00 | 4,53 | 3,31 | 3,44 | 3,33 | 2,58 | 4 | 10 | 4,50 | 4,00 | 3,50 | 3,00 | 3,00 | 3 | 0 | 0 |
| ## | 1 | 24 | 2,60 | 4,13 | 3,38 | 2,78 | 3,11 | 2,60 | 0 | 12 | 3,00 | 3,00 | 3,00 | 3,50 | 3,00 | 3 | 0 | 0 |
| ## | 1 | 28 | 3,00 | 2,47 | 3,77 | 3,44 | 3,44 | 2,89 | 1 | 14 | 2,50 | 4,00 | 2,50 | 5,00 | 3,00 | 3 | 0 | 0 |
| ## | 2 | 24 | 3,00 | 2,80 | 2,85 | 3,56 | 3,33 | 2,85 | 15 | 13 | 2,50 | 2,00 | 1,50 | 2,00 | 3,00 | 3 | 0 | 0 |
| ## | 2 | 23 | 2,50 | 2,67 | 3,92 | 3,56 | 3,56 | 2,67 | 11 | 16 | 2,50 | 5,00 | 3,50 | 4,00 | 2,00 | 3 | 0 | 0 |
| ## | 2 | 23 | 2,30 | 3,80 | 4,23 | 5,00 | 3,00 | 1,61 | 6 | 18 | 3,00 | 5,00 | 3,00 | 3,00 | 2,50 | 3 | 0 | 0 |
| ## | 1 | 28 | 2,40 | 4,13 | 3,00 | 2,33 | 2,89 | 2,89 | 4 | 14 | 2,00 | 5,00 | 3,00 | 4,00 | 3,00 | 2 | 1 | 2 |
| ## | 1 | 27 | 3,10 | 4,73 | 3,31 | 3,44 | 3,44 | 2,76 | 2 | 15 | 3,00 | 4,00 | 2,00 | 5,00 | 2,50 | 3 | 1 | 2 |
| ## | 2 | 24 | 3,10 | 4,00 | 3,77 | 3,33 | 3,00 | 2,29 | 6 | 15 | 2,50 | 2,00 | 1,50 | 5,00 | 2,00 | 4 | 1 | 2 |
| ## | 2 | 53 | 3,80 | 2,87 | 3,38 | 3,00 | 2,89 | 3,06 | 5 | 19 | 4,00 | 4,00 | 3,50 | 3,50 | 2,50 | 3 | 0 | 0 |
| ## | 2 | 63 | 2,00 | 3,33 | 2,92 | 3,44 | 1,78 | 1,70 | 2 | 19 | 4,00 | 4,50 | 3,00 | 4,00 | 3,00 | 3 | 1 | 2 |
| ## | 2 | 28 | 2,60 | 3,60 | 3,23 | 3,78 | 3,56 | 2,45 | 6 | 18 | 3,00 | 5,00 | 2,50 | 2,50 | 2,50 | 3 | 0 | 0 |
| ## | 1 | 21 | 2,30 | 4,07 | 3,00 | 2,78 | 4,11 | 3,36 | 0 | 14 | 5,00 | 5,00 | 3,00 | 5,00 | 4,00 | 3 | 0 | 0 |
| ## | 1 | 22 | 2,30 | 4,40 | 2,77 | 2,67 | 3,78 | 3,29 | 0 | 15 | 3,00 | 3,50 | 1,50 | 5,00 | 3,00 | 3 | 0 | 0 |
| ## | 1 | 24 | 1,80 | 4,33 | 2,62 | 2,44 | 3,67 | 3,39 | 4 | 14 | 2,50 | 3,50 | 4,00 | 4,50 | 2,50 | 3 | 0 | 0 |
| ## | 1 | 23 | 2,30 | 4,40 | 3,00 | 2,78 | 4,00 | 3,31 | 0 | 17 | 3,00 | 4,00 | 1,50 | 4,00 | 3,00 | 3 | 0 | 0 |
| ## | 1 | 28 | 3,10 | 3,13 | 3,69 | 3,56 | 3,22 | 2,46 | 1 | 19 | 3,00 | 4,00 | 3,00 | 4,50 | 3,50 | 3 | 0 | 0 |
| ## | 1 | 58 | 2,90 | 2,60 | 3,62 | 4,11 | 2,89 | 2,22 | 1 | 19 | 4,00 | 4,00 | 2,50 | 3,50 | 3,00 | 2 | 0 | 0 |
| ## | 2 | 61 | 2,80 | 2,53 | 3,85 | 4,11 | 3,00 | 2,26 | 2 | 20 | 3,50 | 4,50 | 3,00 | 4,00 | 2,50 | 1 | 0 | 0 |
| ## | 1 | 58 | 3,00 | 2,47 | 4,08 | 4,78 | 2,78 | 2,20 | 3 | 20 | 4,50 | 5,00 | 1,00 | 5,00 | 2,00 | 1 | 0 | 0 |

| | | | | | | | | | | | | | | | | | | |
|---|---|---|---|---|---|---|---|---|---|---|---|---|---|---|---|---|---|---|
| ## | 1 | 39 | 2,70 | 2,33 | 3,77 | 4,11 | 1,89 | 1,86 | 5 | 17 | 3,50 | 3,50 | 3,00 | 3,00 | 3,00 | 4 | 1 | 1 |
| ## | 2 | 31 | 2,50 | 3,13 | 3,08 | 3,56 | 2,67 | 2,05 | 16 | 15 | 4,00 | 4,00 | 3,50 | 3,00 | 4,00 | 4 | 1 | 1 |
| ## | 2 | 39 | 2,90 | 3,07 | 2,92 | 3,11 | 2,67 | 2,52 | 3 | 18 | 3,00 | 2,50 | 3,50 | 1,00 | 3,00 | 2 | 0 | 0 |
| ## | 1 | 40 | 1,70 | 2,53 | 3,38 | 3,44 | 1,89 | 1,95 | 1 | 16 | 3,50 | 4,50 | 4,00 | 3,50 | 2,50 | 3 | 0 | 0 |
| ## | 2 | 30 | 3,40 | 2,87 | 3,85 | 4,67 | 2,22 | 1,93 | 15 | 15 | 3,50 | 3,50 | 2,50 | 4,00 | 4,50 | 2 | 1 | 1 |
| ## | 2 | 26 | 2,80 | 2,73 | 3,08 | 3,22 | 2,33 | 2,35 | 1 | 19 | 2,50 | 1,50 | 3,00 | 4,00 | 3,00 | 2 | 1 | 2 |
| ## | 1 | 53 | 1,90 | 2,80 | 2,92 | 2,89 | 2,67 | 2,58 | 1 | 17 | 3,50 | 2,00 | 3,00 | 3,00 | 3,00 | 0 | 0 | 0 |
| ## | 2 | 26 | 3,50 | 3,27 | 3,85 | 3,00 | 3,00 | 2,76 | 2 | 15 | 4,00 | 5,00 | 2,50 | 3,50 | 3,50 | 1 | 1 | 1 |
| ## | 2 | 59 | 1,80 | 1,87 | 3,77 | 4,00 | 1,78 | 2,16 | 12 | 17 | 4,00 | 2,50 | 4,00 | 2,50 | 4,50 | 1 | 0 | 0 |
| ## | 2 | 25 | 2,70 | 3,13 | 3,38 | 3,89 | 3,00 | 2,07 | 0 | 17 | 3,00 | 3,50 | 2,50 | 3,50 | 3,50 | 3 | 0 | 0 |
| ## | 2 | 20 | 2,80 | 3,47 | 3,38 | 3,78 | 3,22 | 2,22 | 0 | 17 | 3,00 | 5,00 | 3,50 | 5,00 | 4,00 | 3 | 1 | 1 |
| ## | 2 | 22 | 3,80 | 3,60 | 3,85 | 3,78 | 3,78 | 3,07 | 0 | 15 | 3,00 | 4,00 | 3,00 | 5,00 | 4,00 | 3 | 0 | 0 |
| ## | 1 | 20 | 3,60 | 3,40 | 3,46 | 4,22 | 3,56 | 2,76 | 1 | 14 | 3,00 | 4,00 | 3,00 | 4,00 | 3,50 | 1 | 0 | 0 |
| ## | 2 | 26 | 2,60 | 3,33 | 3,31 | 3,67 | 3,00 | 2,08 | 0 | 19 | 2,00 | 5,00 | 4,00 | 5,00 | 3,00 | 3 | 0 | 0 |
| ## | 2 | 24 | 2,60 | 3,13 | 3,46 | 3,67 | 3,00 | 2,10 | 0 | 17 | 3,00 | 3,50 | 3,00 | 3,50 | 3,50 | 0 | 0 | 0 |
| ## | 1 | 27 | 2,90 | 3,73 | 3,62 | 3,89 | 3,33 | 2,22 | 1 | 18 | 2,00 | 5,00 | 3,00 | 5,00 | 4,00 | 4 | 1 | 1 |
| ## | 1 | 25 | 3,10 | 3,67 | 3,77 | 3,78 | 3,56 | 2,52 | 0 | 16 | 2,50 | 4,00 | 2,50 | 3,50 | 3,00 | 3 | 0 | 0 |
| ## | 2 | 26 | 2,90 | 3,80 | 3,69 | 3,89 | 2,67 | 1,70 | 0 | 13 | 2,50 | 2,50 | 3,00 | 2,00 | 2,50 | 3 | 0 | 0 |
| ## | 1 | 47 | 2,50 | 2,80 | 3,54 | 3,78 | 2,89 | 2,08 | 2 | 18 | 3,00 | 4,00 | 2,50 | 4,50 | 3,00 | 2 | 0 | 0 |
| ## | 1 | 45 | 2,80 | 2,93 | 3,69 | 4,22 | 2,78 | 1,88 | 0 | 20 | 2,50 | 3,50 | 3,00 | 3,00 | 3,00 | 2 | 0 | 0 |
| ## | 2 | 45 | 2,70 | 2,93 | 3,46 | 4,33 | 2,78 | 1,87 | 3 | 21 | 2,00 | 5,00 | 1,00 | 5,00 | 3,00 | 3 | 0 | 0 |
| ## | 1 | 23 | 2,80 | 3,33 | 3,77 | 3,33 | 3,11 | 2,30 | 0 | 15 | 3,00 | 3,50 | 2,50 | 4,00 | 3,50 | 1 | 0 | 0 |
| ## | 2 | 31 | 3,10 | 4,33 | 4,00 | 3,78 | 3,56 | 2,53 | 0 | 18 | 2,50 | 4,50 | 2,50 | 5,00 | 4,00 | 3 | 0 | 0 |
| ## | 2 | 26 | 2,80 | 3,53 | 4,08 | 3,56 | 2,56 | 1,75 | 0 | 17 | 2,50 | 4,00 | 2,00 | 5,00 | 4,00 | 4 | 1 | 1 |
| ## | 2 | 25 | 3,10 | 3,20 | 3,31 | 3,44 | 3,11 | 2,49 | 1 | 21 | 2,00 | 5,00 | 2,00 | 4,50 | 4,50 | 4 | 0 | 0 |
| ## | 2 | 25 | 2,90 | 3,47 | 3,69 | 3,44 | 3,44 | 2,51 | 0 | 16 | 2,50 | 3,50 | 3,50 | 3,50 | 3,00 | 3 | 0 | 0 |
| ## | 1 | 30 | 3,30 | 3,73 | 3,69 | 3,33 | 3,33 | 2,63 | 0 | 20 | 2,50 | 4,50 | 1,50 | 4,50 | 4,00 | 3 | 0 | 0 |
| ## | 2 | 24 | 3,10 | 4,13 | 4,00 | 3,78 | 3,22 | 2,24 | 0 | 21 | 3,50 | 4,00 | 1,50 | 3,50 | 4,00 | 3 | 0 | 0 |
| ## | 2 | 23 | 3,00 | 3,73 | 3,31 | 3,67 | 3,22 | 2,33 | 0 | 17 | 3,00 | 4,00 | 3,00 | 4,00 | 4,00 | 3 | 0 | 0 |
| ## | 1 | 23 | 3,00 | 3,87 | 3,85 | 3,89 | 3,44 | 2,33 | 1 | 11 | 2,00 | 5,00 | 1,00 | 5,00 | 4,00 | 4 | 0 | 0 |
| ## | 1 | 28 | 3,10 | 3,67 | 3,54 | 3,67 | 3,00 | 2,17 | 0 | 14 | 2,50 | 3,50 | 3,00 | 4,00 | 3,00 | 3 | 0 | 0 |
| ## | 1 | 28 | 3,20 | 4,07 | 3,85 | 3,78 | 3,56 | 2,55 | 1 | 18 | 2,00 | 4,00 | 1,00 | 4,00 | 3,00 | 3 | 0 | 0 |
| ## | 1 | 29 | 3,50 | 3,60 | 3,54 | 3,56 | 3,56 | 2,83 | 0 | 18 | 1,00 | 5,00 | 1,00 | 5,00 | 4,00 | 4 | 0 | 0 |

| | | | | | | | | | | | | | | | | | | |
|---|---|---|---|---|---|---|---|---|---|---|---|---|---|---|---|---|---|---|
| ## | 1 | 33 | 1,00 | 4,00 | 2,46 | 4,44 | 2,33 | 2,00 | 3 | 18 | 2,50 | 3,00 | 3,50 | 4,00 | 3,00 | 4 | 0 | 0 |
| ## | 2 | 57 | 2,70 | 2,80 | 3,00 | 3,44 | 2,78 | 2,40 | 1 | 14 | 4,00 | 2,50 | 3,00 | 3,50 | 3,00 | 0 | 0 | 0 |
| ## | 2 | 26 | 1,80 | 3,13 | 3,62 | 3,33 | 2,11 | 1,65 | 4 | 20 | 4,50 | 3,00 | 4,50 | 3,00 | 3,00 | 3 | 0 | 0 |
| ## | 1 | 22 | 2,40 | 3,33 | 4,46 | 3,89 | 2,78 | 1,70 | 0 | 16 | 4,00 | 5,00 | 3,00 | 4,00 | 4,00 | 3 | 0 | 0 |
| ## | 2 | 25 | 2,80 | 3,60 | 3,77 | 3,44 | 3,22 | 2,27 | 0 | 18 | 5,00 | 5,00 | 3,50 | 5,00 | 3,50 | 4 | 0 | 0 |
| ## | 2 | 23 | 2,40 | 3,47 | 3,85 | 3,89 | 3,11 | 1,87 | 1 | 16 | 4,00 | 4,00 | 3,50 | 5,00 | 3,50 | 4 | 0 | 0 |
| ## | 2 | 53 | 3,10 | 2,47 | 3,31 | 3,89 | 3,11 | 2,64 | 13 | 18 | 3,00 | 5,00 | 1,50 | 5,00 | 2,50 | 1 | 0 | 0 |
| ## | 2 | 46 | 3,50 | 3,47 | 3,92 | 5,00 | 4,22 | 3,19 | 7 | 15 | 2,00 | 3,00 | 2,00 | 2,50 | 3,00 | 3 | 0 | 0 |
| ## | 2 | 19 | 3,20 | 3,13 | 3,38 | 3,11 | 2,78 | 2,53 | 16 | 17 | 3,00 | 4,00 | 3,50 | 3,50 | 3,00 | 2 | 0 | 0 |
| ## | 2 | 27 | 2,80 | 3,67 | 3,85 | 3,67 | 2,00 | 1,39 | 12 | 13 | 3,00 | 4,50 | 3,50 | 3,00 | 4,50 | 3 | 0 | 0 |
| ## | 2 | 22 | 2,90 | 3,13 | 2,77 | 3,11 | 2,67 | 2,57 | 7 | 15 | 2,00 | 5,00 | 2,50 | 2,00 | 1,00 | 2 | 0 | 0 |
| ## | 2 | 26 | 2,90 | 3,87 | 4,15 | 3,78 | 2,89 | 1,88 | 0 | 16 | 4,50 | 3,50 | 4,00 | 4,50 | 3,00 | 4 | 0 | 0 |
| ## | 1 | 47 | 3,00 | 2,87 | 3,69 | 3,67 | 2,78 | 2,18 | 7 | 17 | 4,00 | 4,00 | 1,00 | 4,00 | 1,00 | 3 | 0 | 0 |
| ## | 2 | 35 | 2,80 | 3,80 | 3,69 | 3,44 | 3,33 | 2,35 | 0 | 19 | 5,00 | 3,50 | 2,50 | 4,50 | 1,50 | 3 | 0 | 0 |
| ## | 2 | 27 | 2,80 | 3,67 | 3,54 | 3,44 | 3,22 | 2,30 | 0 | 18 | 4,00 | 3,00 | 3,50 | 4,00 | 2,50 | 4 | 0 | 0 |
| ## | 2 | 31 | 2,80 | 3,87 | 3,69 | 3,56 | 3,11 | 2,12 | 0 | 17 | 4,50 | 4,00 | 3,00 | 4,50 | 3,50 | 4 | 1 | 1 |
| ## | 2 | 29 | 2,90 | 3,87 | 3,85 | 3,56 | 3,44 | 2,41 | 0 | 16 | 4,00 | 4,00 | 3,00 | 3,50 | 3,50 | 0 | 0 | 0 |
| ## | 2 | 28 | 1,90 | 3,13 | 3,00 | 3,33 | 2,33 | 1,97 | 6 | 17 | 3,50 | 3,50 | 4,00 | 3,50 | 2,50 | 4 | 0 | 0 |
| ## | 1 | 26 | 2,80 | 3,93 | 3,62 | 3,67 | 3,11 | 2,08 | 3 | 18 | 2,50 | 4,50 | 3,50 | 4,50 | 4,00 | 4 | 0 | 0 |
| ## | 2 | 46 | 2,80 | 3,87 | 3,85 | 3,78 | 3,22 | 2,10 | 0 | 19 | 4,50 | 5,00 | 3,50 | 5,00 | 4,00 | 3 | 0 | 0 |
| ## | 1 | 25 | 2,70 | 3,40 | 3,85 | 3,44 | 3,00 | 2,10 | 1 | 15 | 3,50 | 3,50 | 2,00 | 5,00 | 3,00 | 4 | 0 | 0 |
| ## | 2 | 36 | 2,80 | 3,60 | 3,62 | 3,33 | 2,67 | 1,98 | 0 | 16 | 3,00 | 3,50 | 3,00 | 4,50 | 4,00 | 4 | 1 | 2 |
| ## | 1 | 21 | 2,70 | 3,47 | 3,85 | 3,56 | 2,89 | 1,95 | 0 | 13 | 2,50 | 5,00 | 3,50 | 5,00 | 3,00 | 3 | 0 | 0 |
| ## | 2 | 26 | 2,70 | 3,93 | 3,62 | 3,67 | 3,11 | 2,04 | 1 | 14 | 3,00 | 4,50 | 3,50 | 4,00 | 4,00 | 4 | 0 | 0 |
| ## | 1 | 29 | 2,90 | 3,93 | 3,85 | 3,67 | 3,11 | 2,10 | 2 | 18 | 4,00 | 3,50 | 3,50 | 4,00 | 3,00 | 4 | 0 | 0 |
| ## | 1 | 23 | 2,60 | 4,00 | 3,77 | 3,67 | 3,00 | 1,90 | 0 | 15 | 2,00 | 3,00 | 2,00 | 5,00 | 4,00 | 4 | 0 | 0 |
| ## | 1 | 25 | 2,80 | 3,60 | 3,62 | 3,44 | 3,00 | 2,13 | 0 | 15 | 3,50 | 4,00 | 1,50 | 4,50 | 3,50 | 4 | 0 | 0 |
| ## | 1 | 23 | 2,90 | 4,07 | 4,00 | 3,78 | 3,22 | 2,14 | 0 | 14 | 2,50 | 4,00 | 2,50 | 3,00 | 3,50 | 3 | 0 | 0 |
| ## | 2 | 24 | 2,80 | 4,00 | 3,85 | 3,78 | 3,33 | 2,19 | 0 | 19 | 3,00 | 4,00 | 1,50 | 4,00 | 3,50 | 4 | 0 | 0 |
| ## | 1 | 27 | 2,70 | 3,87 | 4,08 | 3,56 | 3,00 | 1,98 | 0 | 17 | 3,00 | 4,00 | 2,50 | 4,50 | 3,50 | 3 | 0 | 0 |
| ## | 2 | 28 | 2,80 | 3,73 | 3,69 | 3,56 | 3,11 | 2,13 | 0 | 17 | 4,00 | 3,00 | 3,50 | 4,00 | 3,00 | 3 | 0 | 0 |
| ## | 1 | 30 | 2,50 | 2,73 | 3,15 | 3,56 | 2,22 | 2,00 | 7 | 17 | 2,50 | 3,50 | 2,50 | 2,00 | 4,50 | 4 | 1 | 3 |
| ## | 2 | 31 | 4,40 | 3,73 | 3,15 | 4,11 | 4,00 | 3,64 | 9 | 17 | 3,50 | 4,50 | 1,00 | 2,50 | 4,50 | 3 | 1 | 2 |

| | | | | | | | | | | | | | | | | | | |
|---|---|---|---|---|---|---|---|---|---|---|---|---|---|---|---|---|---|---|
| ## | 2 | 30 | 3,60 | 2,87 | 3,62 | 4,00 | 4,00 | 3,25 | 6 | 16 | 2,50 | 3,00 | 1,50 | 3,50 | 4,00 | 4 | 1 | 2 |
| ## | 1 | 37 | 3,50 | 3,87 | 3,54 | 4,56 | 2,67 | 1,99 | 8 | 20 | 2,00 | 3,00 | 2,50 | 3,00 | 2,00 | 3 | 1 | 2 |
| ## | 2 | 35 | 3,10 | 3,47 | 3,62 | 3,44 | 3,22 | 2,44 | 10 | 17 | 4,00 | 3,00 | 1,50 | 4,50 | 2,50 | 2 | 0 | 0 |
| ## | 1 | 30 | 2,70 | 3,20 | 3,69 | 3,33 | 3,00 | 2,24 | 0 | 16 | 2,50 | 5,00 | 5,00 | 4,00 | 1,00 | 2 | 0 | 0 |
| ## | 1 | 24 | 2,80 | 3,60 | 3,62 | 3,11 | 3,00 | 2,33 | 2 | 15 | 2,00 | 4,00 | 3,00 | 4,00 | 3,50 | 3 | 1 | 2 |
| ## | 1 | 24 | 2,80 | 4,27 | 3,23 | 3,22 | 3,44 | 2,67 | 0 | 15 | 3,50 | 3,50 | 3,00 | 4,50 | 3,00 | 3 | 1 | 1 |
| ## | 1 | 24 | 2,50 | 3,27 | 4,23 | 4,33 | 2,89 | 1,66 | 0 | 15 | 4,00 | 5,00 | 4,50 | 4,00 | 3,00 | 4 | 0 | 0 |
| ## | 1 | 24 | 2,20 | 3,87 | 2,00 | 2,78 | 3,22 | 3,22 | 3 | 10 | 3,00 | 3,00 | 1,00 | 5,00 | 2,50 | 3 | 0 | 0 |
| ## | 1 | 29 | 2,40 | 3,27 | 3,62 | 3,67 | 3,11 | 2,05 | 0 | 17 | 4,00 | 4,50 | 3,00 | 3,50 | 3,00 | 4 | 1 | 1 |
| ## | 2 | 25 | 2,60 | 3,67 | 3,54 | 4,11 | 3,22 | 1,97 | 0 | 12 | 4,50 | 4,50 | 3,50 | 3,50 | 3,00 | 4 | 1 | 2 |
| ## | 2 | 23 | 2,40 | 3,27 | 3,62 | 3,56 | 3,11 | 2,11 | 0 | 16 | 4,00 | 4,50 | 3,50 | 3,50 | 3,00 | 4 | 0 | 0 |
| ## | 2 | 24 | 2,50 | 3,33 | 3,62 | 3,56 | 3,11 | 2,11 | 0 | 16 | 4,00 | 4,50 | 3,00 | 3,50 | 3,50 | 3 | 0 | 0 |
| ## | 1 | 37 | 2,70 | 3,60 | 3,23 | 2,78 | 2,56 | 2,38 | 9 | 15 | 2,00 | 3,00 | 2,50 | 2,00 | 2,00 | 3 | 1 | 1 |
| ## | 2 | 54 | 2,80 | 2,93 | 3,23 | 3,11 | 3,00 | 2,59 | 13 | 18 | 3,50 | 4,00 | 3,50 | 2,50 | 3,00 | 3 | 0 | 0 |
| ## | 2 | 59 | 2,10 | 1,80 | 3,23 | 3,22 | 1,78 | 2,65 | 2 | 19 | 3,50 | 3,50 | 4,50 | 3,00 | 3,00 | 2 | 0 | 0 |
| ## | 2 | 54 | 2,30 | 2,00 | 3,00 | 3,33 | 2,22 | 2,62 | 15 | 16 | 2,50 | 3,00 | 2,50 | 3,00 | 1,50 | 2 | 0 | 0 |
| ## | 1 | 32 | 2,50 | 3,07 | 2,92 | 3,11 | 2,78 | 2,45 | 3 | 19 | 3,50 | 4,00 | 3,50 | 2,50 | 3,00 | 3 | 0 | 0 |
| ## | 2 | 70 | 2,90 | 2,60 | 3,15 | 2,78 | 2,78 | 2,87 | 13 | 19 | 3,00 | 3,50 | 3,50 | 2,50 | 3,00 | 0 | 0 | 0 |
| ## | 2 | 28 | 2,70 | 3,07 | 2,62 | 2,89 | 3,00 | 2,89 | 11 | 17 | 3,50 | 4,00 | 2,50 | 2,50 | 3,00 | 3 | 1 | 2 |
| ## | 2 | 53 | 3,10 | 2,73 | 4,08 | 4,33 | 2,89 | 2,17 | 0 | 21 | 3,50 | 3,50 | 2,00 | 2,50 | 3,50 | 3 | 0 | 0 |
| ## | 1 | 50 | 2,80 | 2,13 | 3,77 | 4,11 | 3,11 | 2,59 | 3 | 20 | 3,00 | 4,50 | 3,00 | 2,50 | 3,50 | 3 | 0 | 0 |
| ## | 1 | 50 | 2,80 | 2,73 | 3,15 | 3,56 | 3,00 | 2,48 | 2 | 20 | 3,00 | 4,50 | 2,50 | 4,00 | 2,50 | 1 | 0 | 0 |
| ## | 2 | 36 | 2,30 | 2,93 | 3,69 | 3,56 | 2,78 | 1,97 | 0 | 17 | 4,50 | 5,00 | 2,00 | 4,50 | 4,00 | 3 | 0 | 0 |
| ## | 2 | 21 | 2,60 | 3,27 | 4,31 | 3,89 | 2,78 | 1,75 | 0 | 20 | 2,00 | 5,00 | 3,00 | 5,00 | 4,00 | 3 | 1 | 2 |
| ## | 1 | 22 | 2,40 | 3,33 | 3,69 | 3,22 | 3,00 | 2,18 | 0 | 14 | 2,00 | 5,00 | 3,50 | 4,50 | 4,00 | 3 | 0 | 0 |
| ## | 1 | 25 | 1,90 | 4,33 | 4,15 | 4,11 | 3,00 | 1,64 | 0 | 14 | 2,00 | 5,00 | 1,00 | 5,00 | 4,00 | 3 | 0 | 0 |
| ## | 2 | 28 | 2,40 | 2,80 | 3,54 | 3,22 | 2,67 | 2,21 | 0 | 20 | 3,00 | 5,00 | 4,00 | 5,00 | 4,00 | 3 | 1 | 2 |
| ## | 1 | 28 | 2,60 | 3,27 | 3,54 | 3,33 | 3,00 | 2,21 | 0 | 19 | 3,00 | 4,00 | 1,50 | 5,00 | 3,00 | 3 | 0 | 0 |
| ## | 1 | 30 | 2,50 | 3,33 | 3,69 | 3,56 | 2,89 | 1,93 | 0 | 12 | 3,00 | 4,00 | 1,00 | 5,00 | 4,00 | 3 | 0 | 0 |
| ## | 1 | 28 | 4,60 | 2,60 | 3,23 | 4,33 | 4,00 | 3,95 | 7 | 20 | 3,00 | 3,00 | 3,00 | 1,00 | 5,00 | 2 | 0 | 0 |
| ## | 1 | 57 | 2,80 | 3,80 | 3,23 | 3,00 | 3,33 | 2,69 | 4 | 21 | 4,00 | 3,00 | 3,00 | 3,00 | 3,50 | 2 | 1 | 2 |
| ## | 2 | 70 | 2,50 | 1,80 | 2,77 | 3,11 | 2,00 | 2,95 | 19 | 19 | 3,00 | 3,00 | 3,50 | 3,50 | 2,00 | 2 | 0 | 0 |
| ## | 1 | 19 | 1,60 | 4,73 | 2,08 | 2,22 | 3,67 | 3,86 | 1 | 11 | 3,00 | 3,50 | 4,00 | 5,00 | 3,50 | 3 | 0 | 0 |

| | | | | | | | | | | | | | | | | | | |
|---|---|---|---|---|---|---|---|---|---|---|---|---|---|---|---|---|---|---|
| ## | 2 | 46 | 3,70 | 3,07 | 3,46 | 3,33 | 3,33 | 3,00 | 9 | 18 | 3,50 | 5,00 | 3,00 | 5,00 | 3,50 | 2 | 0 | 0 |
| ## | 1 | 46 | 2,60 | 2,53 | 3,15 | 3,33 | 2,89 | 2,56 | 15 | 12 | 3,00 | 3,00 | 3,00 | 3,00 | 3,00 | 2 | 0 | 0 |
| ## | 2 | 29 | 2,80 | 2,40 | 3,77 | 1,67 | 1,56 | 3,41 | 0 | 15 | 1,00 | 5,00 | 3,00 | 5,00 | 5,00 | 4 | 0 | 0 |
| ## | 2 | 29 | 3,30 | 3,80 | 2,92 | 2,67 | 2,78 | 2,89 | 1 | 9 | 3,00 | 5,00 | 4,50 | 5,00 | 4,50 | 3 | 0 | 0 |
| ## | 2 | 54 | 2,00 | 2,73 | 3,23 | 4,33 | 4,00 | 2,88 | 1 | 19 | 3,50 | 5,00 | 3,00 | 3,50 | 1,50 | 1 | 0 | 0 |
| ## | 1 | 52 | 2,70 | 2,53 | 3,15 | 4,22 | 3,33 | 2,58 | 2 | 21 | 4,00 | 5,00 | 2,00 | 4,50 | 1,50 | 2 | 0 | 0 |
| ## | 2 | 30 | 3,10 | 3,53 | 3,92 | 3,89 | 2,33 | 1,63 | 3 | 16 | 1,50 | 4,00 | 3,00 | 4,50 | 3,00 | 3 | 0 | 0 |
| ## | 2 | 22 | 1,50 | 3,47 | 3,54 | 3,89 | 2,44 | 1,41 | 5 | 12 | 4,00 | 3,00 | 4,00 | 4,00 | 3,50 | 4 | 0 | 0 |
| ## | 2 | 28 | 3,30 | 3,60 | 3,54 | 3,67 | 3,22 | 2,44 | 7 | 9 | 2,00 | 4,00 | 2,50 | 3,50 | 2,50 | 4 | 0 | 0 |
| ## | 2 | 22 | 1,80 | 2,47 | 3,92 | 4,33 | 1,22 | 1,49 | 3 | 16 | 2,00 | 4,00 | 3,00 | 2,00 | 5,00 | 4 | 0 | 0 |
| ## | 1 | 28 | 3,40 | 2,53 | 3,69 | 3,67 | 2,33 | 2,37 | 6 | 25 | 3,00 | 5,00 | 3,50 | 4,00 | 1,50 | 1 | 0 | 0 |
| ## | 2 | 57 | 2,60 | 1,93 | 2,62 | 4,00 | 2,11 | 2,63 | 8 | 17 | 1,50 | 1,00 | 2,00 | 3,50 | 5,00 | 2 | 0 | 0 |
| ## | 1 | 21 | 1,50 | 2,27 | 3,31 | 3,67 | 1,78 | 2,07 | 4 | 18 | 4,50 | 4,50 | 5,00 | 5,00 | 2,50 | 3 | 1 | 2 |
| ## | 2 | 18 | 3,60 | 3,73 | 3,69 | 4,00 | 3,44 | 2,64 | 3 | 17 | 4,50 | 5,00 | 2,50 | 3,00 | 2,00 | 2 | 0 | 0 |
| ## | 1 | 56 | 3,70 | 2,67 | 2,08 | 3,44 | 2,00 | 3,14 | 5 | 16 | 4,50 | 4,50 | 3,50 | 2,00 | 5,00 | 3 | 1 | 3 |
| ## | 1 | 32 | 2,00 | 3,20 | 2,85 | 4,33 | 2,56 | 1,73 | 4 | 13 | 4,00 | 3,50 | 4,00 | 5,00 | 3,00 | 3 | 1 | 2 |
| ## | 1 | 61 | 2,00 | 3,40 | 2,23 | 2,89 | 2,78 | 2,82 | 0 | 18 | 4,50 | 3,50 | 5,00 | 3,00 | 3,00 | 2 | 0 | 0 |
| ## | 1 | 28 | 3,20 | 3,73 | 3,38 | 4,22 | 2,89 | 2,01 | 7 | 16 | 4,00 | 4,00 | 5,00 | 3,00 | 4,00 | 4 | 0 | 0 |
| ## | 2 | 41 | 3,90 | 2,47 | 2,85 | 3,78 | 3,33 | 3,35 | 13 | 18 | 5,00 | 2,00 | 1,00 | 2,50 | 3,00 | 2 | 0 | 0 |
| ## | 1 | 28 | 1,70 | 2,53 | 3,77 | 3,78 | 1,11 | 1,68 | 0 | 17 | 3,00 | 3,00 | 4,50 | 3,50 | 4,50 | 3 | 1 | 2 |
| ## | 1 | 28 | 3,20 | 3,53 | 3,77 | 3,67 | 3,11 | 2,28 | 13 | 12 | 3,50 | 1,50 | 3,00 | 2,50 | 3,50 | 2 | 0 | 0 |
| ## | 1 | 34 | 1,80 | 3,93 | 2,08 | 2,56 | 2,67 | 3,04 | 1 | 11 | 4,00 | 3,50 | 3,00 | 5,00 | 3,00 | 3 | 1 | 1 |
| ## | 2 | 58 | 2,70 | 2,53 | 3,62 | 3,56 | 2,00 | 1,98 | 5 | 19 | 3,00 | 5,00 | 4,00 | 2,50 | 4,00 | 3 | 0 | 0 |
| ## | 2 | 36 | 3,40 | 3,27 | 3,62 | 3,33 | 2,56 | 2,32 | 16 | 13 | 4,00 | 4,00 | 5,00 | 3,00 | 2,50 | 3 | 1 | 3 |
| ## | 2 | 39 | 3,10 | 3,40 | 4,00 | 3,44 | 3,00 | 2,27 | 3 | 17 | 4,50 | 4,00 | 4,00 | 3,00 | 3,00 | 3 | 1 | 2 |
| ## | 2 | 28 | 2,50 | 3,33 | 3,15 | 3,67 | 2,56 | 1,82 | 10 | 13 | 1,50 | 3,00 | 2,00 | 4,00 | 4,00 | 3 | 0 | 0 |
| ## | 2 | 36 | 2,30 | 2,07 | 3,23 | 3,89 | 2,67 | 2,44 | 14 | 14 | 4,00 | 5,00 | 2,50 | 4,00 | 2,00 | 2 | 0 | 0 |
| ## | 2 | 30 | 2,20 | 3,40 | 3,31 | 3,22 | 2,33 | 1,84 | 9 | 15 | 3,50 | 3,50 | 3,00 | 4,00 | 3,50 | 4 | 0 | 0 |
| ## | 2 | 25 | 2,90 | 3,13 | 4,23 | 4,56 | 2,33 | 1,50 | 3 | 16 | 3,00 | 5,00 | 3,00 | 3,00 | 4,00 | 4 | 0 | 0 |
| ## | 2 | 28 | 3,50 | 3,60 | 3,38 | 4,00 | 3,22 | 2,49 | 13 | 17 | 3,50 | 4,00 | 1,50 | 3,00 | 4,00 | 4 | 0 | 0 |
| ## | 2 | 26 | 4,30 | 3,60 | 2,85 | 2,33 | 3,89 | 4,22 | 10 | 16 | 3,50 | 2,50 | 2,00 | 4,00 | 5,00 | 4 | 0 | 0 |
| ## | 2 | 39 | 3,10 | 3,80 | 3,31 | 3,89 | 3,00 | 2,14 | 5 | 19 | 2,00 | 4,50 | 2,00 | 3,00 | 4,50 | 3 | 0 | 0 |
| ## | 2 | 37 | 3,20 | 3,33 | 3,38 | 3,67 | 2,78 | 2,18 | 4 | 18 | 2,00 | 4,50 | 2,00 | 5,00 | 5,00 | 2 | 0 | 0 |

| | | | | | | | | | | | | | | | | | | |
|---|---|---|---|---|---|---|---|---|---|---|---|---|---|---|---|---|---|---|
| ## | 1 | 22 | 2,00 | 4,07 | 4,00 | 3,89 | 2,56 | 1,28 | 0 | 15 | 2,00 | 5,00 | 2,50 | 5,00 | 4,00 | 3 | 0 | 0 |
| ## | 1 | 49 | 2,80 | 2,73 | 3,54 | 4,00 | 2,44 | 1,88 | 0 | 19 | 3,00 | 5,00 | 3,00 | 5,00 | 3,00 | 2 | 0 | 0 |
| ## | 2 | 24 | 2,30 | 3,73 | 3,85 | 3,56 | 3,00 | 1,87 | 0 | 16 | 2,00 | 4,50 | 3,50 | 2,50 | 4,00 | 3 | 0 | 0 |
| ## | 1 | 31 | 2,70 | 3,00 | 3,23 | 3,78 | 2,56 | 1,94 | 0 | 18 | 3,00 | 4,00 | 2,50 | 3,50 | 3,00 | 4 | 0 | 0 |
| ## | 1 | 26 | 2,50 | 3,20 | 3,54 | 3,78 | 2,89 | 1,90 | 0 | 19 | 3,00 | 4,50 | 4,00 | 3,50 | 3,50 | 3 | 0 | 0 |
| ## | 1 | 27 | 2,30 | 3,87 | 4,15 | 4,00 | 2,89 | 1,56 | 0 | 17 | 1,00 | 5,00 | 2,00 | 5,00 | 3,00 | 3 | 0 | 0 |
| ## | 2 | 21 | 1,80 | 2,60 | 3,08 | 4,44 | 2,22 | 1,76 | 0 | 15 | 3,50 | 4,00 | 5,00 | 4,00 | 3,00 | 3 | 0 | 0 |
| ## | 2 | 37 | 2,20 | 2,00 | 3,92 | 4,89 | 1,56 | 1,94 | 3 | 18 | 3,50 | 5,00 | 5,00 | 3,00 | 2,50 | 4 | 0 | 0 |
| ## | 1 | 61 | 2,70 | 2,13 | 3,38 | 3,56 | 2,56 | 2,50 | 1 | 21 | 3,00 | 3,00 | 3,00 | 4,00 | 4,00 | 3 | 0 | 0 |
| ## | 2 | 23 | 2,50 | 3,60 | 4,00 | 3,89 | 2,89 | 1,68 | 0 | 16 | 2,00 | 5,00 | 2,00 | 5,00 | 4,00 | 3 | 0 | 0 |
| ## | 2 | 46 | 2,20 | 3,27 | 3,62 | 4,11 | 3,22 | 1,95 | 0 | 20 | 2,50 | 3,00 | 3,00 | 2,50 | 4,50 | 3 | 0 | 0 |
| ## | 2 | 25 | 2,20 | 3,47 | 3,69 | 3,67 | 2,78 | 1,69 | 0 | 16 | 2,00 | 5,00 | 3,00 | 5,00 | 4,00 | 3 | 0 | 0 |
| ## | 2 | 56 | 2,70 | 2,53 | 3,08 | 3,11 | 2,78 | 2,67 | 1 | 20 | 2,50 | 4,00 | 3,00 | 4,00 | 3,00 | 2 | 0 | 0 |
| ## | 2 | 20 | 3,00 | 4,20 | 3,46 | 3,44 | 3,00 | 2,25 | 5 | 9 | 3,00 | 3,00 | 1,50 | 3,00 | 2,50 | 3 | 0 | 0 |
| ## | 2 | 52 | 1,50 | 3,40 | 3,92 | 4,00 | 3,00 | 1,75 | 4 | 16 | 3,00 | 5,00 | 3,00 | 3,00 | 3,00 | 3 | 0 | 0 |
| ## | 1 | 30 | 2,40 | 2,07 | 2,54 | 3,56 | 2,22 | 2,70 | 7 | 13 | 3,50 | 1,00 | 3,00 | 3,50 | 2,50 | 3 | 0 | 0 |
| ## | 1 | 27 | 2,30 | 2,13 | 2,69 | 3,00 | 2,00 | 2,79 | 9 | 14 | 4,50 | 4,00 | 3,00 | 2,50 | 4,50 | 3 | 1 | 1 |
| ## | 2 | 26 | 2,20 | 3,33 | 3,77 | 3,22 | 2,00 | 1,61 | 16 | 10 | 2,00 | 4,50 | 2,00 | 4,00 | 5,00 | 4 | 1 | 2 |
| ## | 1 | 38 | 2,90 | 3,47 | 3,92 | 3,56 | 2,67 | 1,88 | 0 | 20 | 3,50 | 4,00 | 4,00 | 3,50 | 3,00 | 4 | 0 | 0 |
| ## | 1 | 40 | 4,10 | 3,40 | 3,23 | 3,00 | 3,22 | 3,32 | 23 | 15 | 2,50 | 3,00 | 2,00 | 2,00 | 4,00 | 2 | 0 | 0 |
| ## | 2 | 36 | 3,40 | 3,60 | 4,08 | 3,78 | 2,33 | 1,89 | 0 | 17 | 4,00 | 4,00 | 5,00 | 3,00 | 3,00 | 3 | 0 | 0 |
| ## | 2 | 56 | 3,50 | 3,80 | 3,62 | 3,56 | 3,00 | 2,43 | 1 | 17 | 3,50 | 4,00 | 4,00 | 2,50 | 3,50 | 3 | 0 | 0 |
| ## | 1 | 53 | 3,40 | 3,73 | 3,62 | 3,56 | 3,00 | 2,37 | 4 | 21 | 4,00 | 3,50 | 4,00 | 3,00 | 2,50 | 2 | 0 | 0 |
| ## | 2 | 22 | 3,50 | 4,00 | 3,69 | 3,78 | 2,89 | 2,26 | 6 | 17 | 3,50 | 4,00 | 4,50 | 3,00 | 3,00 | 2 | 1 | 2 |
| ## | 2 | 60 | 3,70 | 3,87 | 3,92 | 4,11 | 3,00 | 2,36 | 6 | 20 | 3,50 | 4,00 | 4,50 | 2,50 | 2,50 | 1 | 1 | 2 |
| ## | 2 | 43 | 2,90 | 3,53 | 3,62 | 3,78 | 3,11 | 2,11 | 2 | 17 | 3,50 | 3,50 | 3,50 | 2,50 | 3,00 | 2 | 0 | 0 |
| ## | 2 | 27 | 4,00 | 3,47 | 3,46 | 3,56 | 3,00 | 2,83 | 19 | 10 | 4,00 | 3,50 | 2,00 | 4,00 | 3,50 | 1 | 0 | 0 |
| ## | 2 | 36 | 3,30 | 3,67 | 3,38 | 3,67 | 3,11 | 2,39 | 6 | 18 | 4,00 | 4,00 | 4,00 | 2,50 | 3,00 | 3 | 0 | 0 |
| ## | 1 | 41 | 3,30 | 3,73 | 3,46 | 3,67 | 3,11 | 2,37 | 6 | 18 | 3,00 | 4,00 | 4,50 | 3,00 | 2,50 | 2 | 0 | 0 |
| ## | 1 | 57 | 3,50 | 3,80 | 3,38 | 3,78 | 3,00 | 2,39 | 5 | 18 | 5,00 | 4,00 | 5,00 | 2,00 | 1,50 | 4 | 0 | 0 |
| ## | 1 | 38 | 3,90 | 3,87 | 3,54 | 3,33 | 2,89 | 2,75 | 3 | 14 | 4,50 | 4,00 | 4,00 | 2,00 | 2,50 | 2 | 0 | 0 |
| ## | 1 | 51 | 3,50 | 3,80 | 3,54 | 3,89 | 3,00 | 2,32 | 0 | 20 | 5,00 | 4,00 | 5,00 | 2,00 | 1,50 | 1 | 0 | 0 |
| ## | 2 | 42 | 3,40 | 3,67 | 3,31 | 3,89 | 3,22 | 2,47 | 1 | 19 | 5,00 | 4,00 | 4,50 | 2,00 | 2,00 | 4 | 0 | 0 |

| | | | | | | | | | | | | | | | | | | | |
|---|---|---|---|---|---|---|---|---|---|---|---|---|---|---|---|---|---|---|---|
| ## | 2 | 39 | 3,60 | 3,80 | 3,46 | 4,22 | 3,11 | 2,40 | 0 | 19 | 4,50 | 3,50 | 5,00 | 2,50 | 1,50 | 3 | 1 | 2 |
| ## | 2 | 47 | 3,70 | 3,60 | 3,54 | 3,67 | 3,00 | 2,55 | 7 | 14 | 5,00 | 4,00 | 4,50 | 2,00 | 2,50 | 2 | 0 | 0 |
| ## | 2 | 39 | 3,90 | 3,73 | 3,69 | 3,00 | 3,78 | 3,42 | 5 | 17 | 4,50 | 3,50 | 4,50 | 2,00 | 2,00 | 3 | 0 | 0 |
| ## | 2 | 31 | 3,40 | 3,80 | 3,46 | 3,89 | 2,89 | 2,20 | 0 | 20 | 5,00 | 4,00 | 5,00 | 2,00 | 1,50 | 2 | 0 | 0 |
| ## | 2 | 36 | 3,60 | 3,87 | 3,62 | 3,67 | 3,00 | 2,45 | 5 | 16 | 4,50 | 3,50 | 5,00 | 2,00 | 1,50 | 2 | 1 | 2 |
